# Disparities in Early Lecanemab Uptake Among US Medicare Beneficiaries

**DOI:** 10.1001/jamanetworkopen.2025.11711

**Published:** 2025-05-15

**Authors:** Frank F. Zhou, Utibe R. Essien, Jeffrey M. Souza, Catherine A. Sarkisian, Cheryl L. Damberg, Bruce E. Landon, John N. Mafi

**Affiliations:** 1 Division of General Internal Medicine and Health Services Research, David Geffen School of Medicine at UCLA, University of California, Los Angeles; 2 Department of Health Care Policy, Harvard Medical School, Boston, Massachusetts; 3 RAND Health, RAND, Santa Monica, California; 4 Division of General Internal Medicine, Beth Israel Deaconess Medical Center, Boston, Massachusetts; 5 Geriatric Research Education and Clinical Center (GRECC), Veterans Association Greater Los Angeles Healthcare System, Los Angeles, California

## Abstract

This cross-sectional study examines lecanemeb update among Medicare fee-for-service beneficiaries with Alzheimer disease or mild cognitive impairment and evaluates whether there are disparities among potentially eligible patients.

## Introduction

In July 2023, lecanemab became the first disease-modifying therapy for Alzheimer disease (AD) to receive broad Medicare coverage. However, patients face significant access barriers, including high out-of-pocket costs, cumbersome testing requirements, and the need for in-person infusions at specialized centers.^1,2,3^ Given that these challenges may be especially experienced by disadvantaged groups disproportionately affected by AD,^4^ we examined early trends in lecanemab uptake among US Medicare beneficiaries, including racial, ethnic, and socioeconomic disparities in its use.

## Methods

In this cross-sectional study, we examined early trends in lecanemab uptake among 100% of Medicare fee-for-service beneficiaries aged 66 years or older with 11 or more months of coverage in the preceding year. Using HCPCS code J0174 in either the Part A or part B claims files, we identified patients who had received at least 1 lecanemab infusion between July 1, 2023, and March 31, 2024, the most recent data available.

We compared the demographics of lecanemab users with all Medicare beneficiaries with diagnosed AD or mild cognitive impairment (MCI), given that all lecanemab users must have either documented AD or MCI for reimbursement, and calculated lecanemab uptake rates by demographic group.^5^ We identified AD using claims containing *International Statistical Classification of Diseases and Related Health Problems, Tenth Revision (ICD-10)* codes G30.0, G30.1, G30.8, or G30.9 in the previous year, and MCI similarly using *ICD-10* codes G31.84 or R41.81.^5^ Sociodemographic variables were drawn from the Centers for Medicare & Medicaid Services Beneficiary Summary File and included age, sex, race and/or ethnicity, urban-rural status, and a proxy variable for socioeconomic disadvantage, defined as being eligible for either the Part D low-income subsidy or dually eligible for Medicare-Medicaid.

We performed χ^2^ testing for between-group differences using SAS Enterprise Guide version 7.15 (SAS Institute) and Excel version 2502 (Microsoft Corp), and considered 2-sided *P* values of less than .01 as significant. We also calculated lecanemab’s discontinuation rate by the end of the study timeframe. This study followed Strengthening the Reporting of Observational Studies in Epidemiology (STROBE) reporting guideline and was approved by the Harvard Medical School institutional review board without need for patient consent given use of deidentified data. Further details are in the eMethods in Supplement 1.

## Results

We identified 1725 Medicare beneficiaries who used lecanemab during our study timeframe (mean [SD] age at initiation, 75.7 [5.2] years); 888 were female (51.5%); by race and ethnicity, 22 were Asian or Pacific Islander (1.3%), 21 Black (1.2%), 35 Hispanic (2.0%), and 1561 were White (90.5%); 1518 (88.0%) were from urban areas, and 23 (1.3%) were socioeconomically disadvantaged (Table). Lecanemab uptake rates among all patients with AD or MCI (842 192 patients) were significantly higher for patients who were male (0.27% vs 0.17% for female; *P* < .001), non-Hispanic White (0.23% vs 0.09% for Asian or Pacific Islander, 0.04% for Black, 0.07% for Hispanic; *P* < .001 for all), from urban areas (0.22% vs 0.14% for rural areas; *P* < .001), or socioeconomically advantaged (0.27% vs 0.01% for socioeconomically disadvantaged; *P* < .001). Results were consistent when analyzing patients with AD and MCI separately. There were 1318 patients using lecanemab in March 2024, suggesting that 407 (23.6%) patients had discontinued lecanemab by this time point (Figure).

**Table.** Baseline Demographics of Patients Who Have Ever Used Lecanemab Compared With Patients With Diagnosed AD or MCI

Demographic variable	Among patients with AD or MCI					Among patients with AD					Among patients with MCI only					
	Lecanemab users, No. (%)	Medicare beneficiaries, No. (%)	Uptake rates of lecanemab, %^a^	χ^2^ (*df*)	*P* value	Lecanemab users, No. (%)	Medicare beneficiaries, No. (%)	Uptake rates of lecanemab, %^a^	χ^2^ (*df*)	*P* value	Lecanemab users, No. (%)	Medicare beneficiaries, No. (%)	Uptake rates of lecanemab, %^a^	χ^2^ (*df*)	*P* value
Total, No.	1725	842 192	0.20	NA	NA	1582	550 945	0.29	NA	NA	143	291 247	0.05	NA	NA
Age, y															
66-69	228 (13.2)	42 869 (5.1)	0.53	1354 (4)	<.001	216 (14.1)	19 201 (3.5)	1.12	1979 (4)	<.001	12 (8.4)	23 668 (8.1)	0.05	39 (4)	<.001
70-74	495 (28.7)	100 572 (11.9)	0.49			462 (29.5)	51 268 (9.3)	0.90			33 (23.1)	49 304 (16.9)	0.07		
75-79	575 (33.3)	160 420 (19.0)	0.37			524 (32.4)	93 850 (17.0)	0.56			51 (35.7)	66 570 (22.9)	0.08		
80-84	333 (19.3)	193 987 (23.0)	0.18			296 (19.0)	129 023 (23.4)	0.23			37 (25.9)	64 964 (22.3)	0.06		
≥85	94 (5.4)	344 344 (40.9)	0.03			84 (5.0)	257 603 (46.8)	0.03			10 (7.0)	86 741 (29.8)	0.01		
Sex															
Female	888 (51.5)	535 645 (63.6)	0.17	110 (1)	<.001	812 (51.6)	366 961 (66.6)	0.22	166 (1)	<.001	76 (53.1)	168 684 (57.9)	0.05	1.3 (1)	.25
Male	837 (48.5)	306 547 (36.4)	0.27			770 (48.4)	183 984 (33.4)	0.42			67 (46.9)	122 563 (42.1)	0.05		
Race/ethnicity^b^															
Asian/Pacific Islander	22 (1.3)	24 973 (3.0)	0.09	192 (4)	<.001	20 (1.4)	17 961 (3.3)	0.11	221 (4)	<.001	2 (1.4)	7012 (2.4)	0.03	14 (4)	.008
Black	21 (1.2)	57 433 (6.8)	0.04			20 (1.4)	42 309 (7.7)	0.05			1 (0.7)	15 124 (5.2)	0.01		
Hispanic	35 (2.0)	47 860 (5.7)	0.07			34 (2.2)	36 406 (6.6)	0.09			1 (0.7)	11 814 (4.1)	0.01		
Non-Hispanic White	1561 (90.5)	690 533 (82.0)	0.23			1430 (90.4)	442 135 (80.3)	0.32			131 (91.6)	248 398 (85.3)	0.05		
Other/unknown	86 (5.0)	21 393 (2.5)	0.40			78 (4.7)	12 494 (2.3)	0.62			8 (5.6)	8899 (3.1)	0.09		
Urban-rural status^c^															
Urban	1518 (88.0)	691 366 (82.1)	0.22	41 (1)	<.001	1392 (88.9)	447 142 (81.2)	0.31	48 (1)	<.001	126 (88.1)	244 224 (83.9)	0.05	2.5 (1)	.12
Rural	206 (11.9)	150 319 (17.8)	0.14			189 (11.1)	103 462 (18.8)	0.18			16 (11.2)	46 857 (16.1)	0.03		
Socioeconomically disadvantaged^d^															
Yes	23 (1.3)	208 374 (24.7)	0.01	508 (1)	<.001	19 (1.2)	164 346 (29.8)	0.01	619 (1)	<.001	4 (2.8)	44 028 (15.1)	0.01	17 (1)	<.001
No	1702 (98.7)	633 818 (75.3)	0.27			1563 (98.8)	386 599 (70.2)	0.40			139 (97.2)	247 219 (84.9)	0.06		
No. of hospitalizations in last 12 mo															
0	1557 (90.3)	536 245 (63.7)	0.29	527 (1)	<.001	1428 (89.1)	321 496 (58.4)	0.44	555 (1)	<.001	129 (90.2)	214 749 (73.7)	0.06	38 (1)	<.001
≥1	168 (9.7)	305 947 (36.3)	0.05			154 (10.9)	189 261 (34.4)	0.08			14 (9.8)	116 686 (40.1)	0.01		
Census region															
Northeast	301 (17.4)	162 701 (19.3)	0.19	146 (3)	<.001	292 (19.2)	105 414 (19.1)	0.28	96 (3)	<.001	9 (6.3)	57 287 (19.7)	0.02	82 (3)	<.001
Midwest	241 (14.0)	165 202 (19.6)	0.15			228 (14.6)	112 146 (20.4)	0.20			13 (9.1)	53 056 (18.2)	0.02		
South	946 (54.8)	345 298 (41.0)	0.27			836 (51.7)	228 289 (41.4)	0.37			110 (76.9)	117 009 (40.2)	0.09		
West	233 (13.5)	166 648 (19.8)	0.14			223 (14.3)	103 360 (18.8)	0.22			10 (7.0)	63 288 (21.7)	0.02		

Abbreviations: AD, Alzheimer dementia; MCI, mild cognitive impairment; NA, not applicable.

^a^ Uptake rates were calculated by dividing the number of lecanemab users by the number of patients with AD or MCI; all lecanemab users also had an associated diagnosis of AD or MCI. If a patient was found to have both a diagnosis of AD and MCI, they were preferentially placed in the AD category.

^b^ Race/ethnicity was defined using the Research Triangle Institute (RTI) Race Code variable. Post-hoc paired comparisons within the race/ethnicity variable confirmed that Non-Hispanic White was significantly different to each of Asian/Pacific Islander, Black, and Hispanic among patients with AD or MCI, with χ^2^ = 21, 90, and 48 respectively, *P* < .001 for all.

^c^ Urban-rural status was defined by converting zip codes to secondary Rural-Urban Commuting Area (RUCA) codes and then using mappings by the Rural Health Research Center.

^d^ The proxy variable for socioeconomic disadvantage was marked as “yes” if the patient was eligible for either the Part D premium low-income subsidy (LIS) or dually eligible for Medicare-Medicaid for ≥1 month during the past 12 months.

**Figure.**
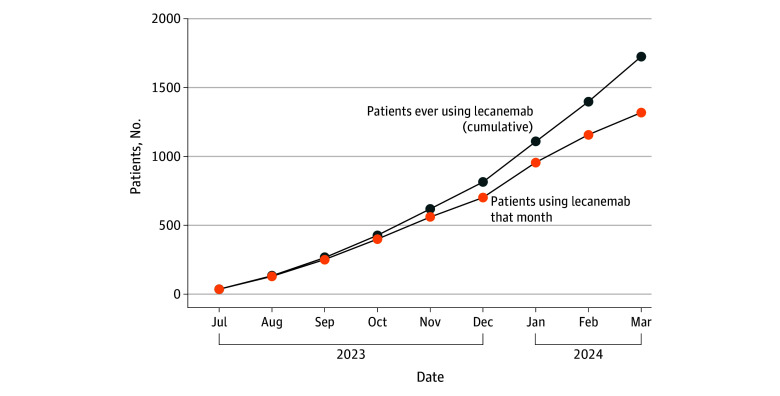
Cumulative and Monthly Users of Lecanemab in the US Medicare Program Monthly and cumulative lecanemab users have grown steadily over time. The discrepancy between monthly and cumulative lecanemab users suggests that 23.6% of users had discontinued lecanemab by March 2024.

## Discussion

Even among beneficiaries who meet initial Medicare coverage requirements for lecanemab by having documented MCI or AD, early uptake of lecanemab still appears to be marked by racial, ethnic, and socioeconomic disparities. This dynamic is consistent with a recurring historical pattern of inequitable access to breakthrough therapies administered by specialized centers,^6^ and underscores how a costly and likely low-value treatment, which contributes to higher Medicare spending, is seemingly being disproportionately utilized by advantaged populations.

This study has limitations. Data for Medicare Advantage beneficiaries were not available. We identified patients with AD or MCI using diagnosis codes, which underestimate MCI prevalence, misdiagnose AD, do not consider additional lecanemab eligibility criteria, and cannot distinguish between mild vs moderate or severe AD, where only the former is eligible for lecanemab. Thus, our uptake rates do not represent uptake among truly eligible patients, but are instead intended for comparing relative uptake among different demographic groups of possibly eligible patients.

## Notes

Supplement 1.
eMethods.

Supplement 2.
Data Sharing Statement
