# Supplementary material for: Disparities in Early Lecanemab Uptake Among US Medicare Beneficiaries
Source: JAMA Netw Open. 2025 May 15;8(5):e2511711. doi: 10.1001/jamanetworkopen.2025.11711 (PMC12082366; doi:10.1001/jamanetworkopen.2025.11711)
Supplement: Supplement 1. — eMethods. [file jamanetwopen-e2511711-s001.pdf]

Supplemental Online Content

Zhou FF, Essien UR, Souza JM, et al. Disparities in early lecanemab uptake among US Medicare beneficiaries. *JAMA Netw Open.* 2025;8(5):e2511711. doi:10.1001/jamanetworkopen.2025.11711

eMethods.

This supplemental material has been provided by the authors to give readers additional information about their work.

© 2025 Zhou FF et al. *JAMA Network Open*.

PATIENT COHORT

We analyzed claims data provided by the Centers for Medicare & Medicaid Services (CMS) for 100% of Medicare fee-for-service beneficiaries aged ≥66 years with at least 11 out of 12 months of Part A or Part B coverage in the preceding year. We excluded Medicare Advantage patients as data was not yet available.

IDENTIFYING PATIENTS WITH DEMENTIA

We classified patients as having Alzheimer's dementia (AD) if they had either of the following during the 12 months prior to the end date of our study timeframe (March 31, 2024):

- One or more inpatient, skilled nursing facility or home health claims with ICD-10 codes G30.0, G30.1, G30.8, or G30.9 as any of the diagnoses on the claim, OR
- Two or more outpatient or carrier claims at least one day apart with the same ICD-10 codes anywhere on the claim.

We classified patients as having mild cognitive impairment (MCI) if they had two claims of any type, at least one day apart, containing codes G31.84 or R41.81 anywhere on the claim during the 12 months prior to the end date of our study timeframe (March 31, 2024).

Our cohort of patients with AD and MCI included everyone meeting the above criteria and did not exclude people who were using lecanemab.

This list of codes was chosen to closely match the codes required by Medicare for lecanemab claims,[1] while also having sufficient sensitivity to identify other patients with diagnosed MCI or AD in the past year.

Our chosen algorithm is comparable to other published algorithms, including the CMS Chronic Conditions Data Warehouse (CCW) algorithm for AD, and the algorithm used by Anderson et al. (2021) for MCI, albeit both with a one-year rather than two-year or three-year lookback respectively to improve specificity.[2,3]

© 2025 Zhou FF et al. *JAMA Network Open*.

IDENTIFYING LECANEMAB USERS

Using HCPCS code J0174 in either the Part A or Part B claims files, we identified patients who had received at least one lecanemab infusion between July 1, 2023 and March 31, 2024, the most recent data available.

For the Table 1 sub-analysis where we looked only among patients with AD or only among patients with MCI, we needed a way to classify lecanemab users as having AD, MCI, or both. To do this, we used the same algorithm as above, except with an adjustment to allow patients to be classified as AD or MCI using only one claim, which could be on the same day as the lecanemab claim. This was because there were a few lecanemab patients whose only documented diagnosis of AD or MCI was on their lecanemab claim, and not on prior visits. If a lecanemab patients was identified as having both AD or MCI, they were classified into the AD group.

DEMOGRAPHIC VARIABLES

Variables were drawn from the CMS Beneficiary Summary File.

Race/ethnicity. This was defined using the Research Triangle Institute (RTI) Race Code variable. The categories were “Asian/Pacific Islander”, “Black (or African American)” (which we renamed to “Black”), “Hispanic”, “Non-Hispanic White” and “Other/unknown” (which includes “American Indian / Alaska Native”).

Socioeconomic disadvantage. This proxy variable for socioeconomic disadvantage was marked as “yes” if the patient was eligible for either the Part D premium low-income subsidy or dually eligible for Medicare-Medicaid for ≥1 month during the past 12 months.

Urban–rural status. This was defined by converting patient zip codes to secondary Rural-Urban Commuting Area (RUCA) codes. We then used the “Categorization D” mapping published by the Rural Health Research Center, reproduced below.[4]

Urban: 1.0, 1.1, 2.0, 2.1, 4.1, 5.1, 7.1, 8.1, and 10.1.

© 2025 Zhou FF et al. *JAMA Network Open*.

Rural: 3.0, 4.0, 4.2, 5.0, 5.2, 6.0, 6.1, 7.0, 7.2, 7.3, 7.4, 8.0, 8.2, 8.3, 8.4, 9.0, 9.1, 9.2, 10.0, 10.2, 10.3, 10.4, 10.5, and 10.6

OUTPUT VARIABLES

Uptake rates were calculated by dividing the number of lecanemab users in each demographic category by the number of patients with AD or MCI in that category. It was confirmed that all lecanemab users also had diagnosed AD or MCI and were thus all were included in the calculations.

These uptake rates should be interpreted with the caveat that many people with AD or MCI may turn out to be ineligible for lecanemab based on clinical or biomarker limitations. For example, lecanemab is indicated for MCI and mild dementia, but not moderate or severe dementia. It is not possible to distinguish between dementia severities using ICD-10 codes, so we were unable to restrict our comparison group to only mild AD. Furthermore, patients only become eligible for lecanemab if they have proven amyloid plaque on PET imaging or CSF studies and do not meet any exclusion criteria. Another caveat is that many patients with MCI cannot be identified from diagnosis codes alone, which is a limitation inherent to all analyses relying on administrative claims. Thus, our uptake rates do not represent uptake among truly eligible patients, but are instead intended for comparing relative lecanemab uptake between different demographic categories and to highlight disparities in lecanemab uptake, which was the primary goal of this analysis.

STATISTICAL TESTING

Chi-square testing was performed on each demographic variable of interest (e.g., age, sex, race/ethnicity, etc.) to determine if the observed numbers of lecanemab users in each of the categories of the demographic variable deviate from expected. Observed numbers of lecanemab users were compared to expected numbers of lecanemab users (assuming users

© 2025 Zhou FF et al. *JAMA Network Open*.

were selected in equal proportion from all patients with AD or MCI). Given that the number of lecanemab users is directly related to uptake rates from the expected population, statistically significant differences in the distribution of lecanemab users also represent statistically significant differences in uptake rates.

In cases of demographic variables with more than two categories (e.g., race/ethnicity), additional direct chi-square tests were performed to confirm differences between pairs of categories. For example, further testing was performed to confirm that “Non-Hispanic White” is significantly different to each of “Asian/Pacific Islander”, “Black” and “Hispanic” among patients with AD or MCI.

© 2025 Zhou FF et al. *JAMA Network Open*.

REFERENCES

1. Centers for Medicare & Medicaid Services. Monoclonal Antibodies Directed Against Amyloid for the Treatment of Alzheimer's Disease (AD) (200.3). Accessed November 14, 2024. https://www.cms.gov/medicare-coverage-database/view/ncd.aspx?ncdid=375

2. Anderson TS, Ayanian JZ, Souza J, Landon BE. Representativeness of Participants Eligible to Be Enrolled in Clinical Trials of Aducanumab for Alzheimer Disease Compared With Medicare Beneficiaries With Alzheimer Disease and Mild Cognitive Impairment. *JAMA*. 2021;326(16):1627-1629. doi:10.1001/jama.2021.15286

3. Centers for Medicare & Medicaid Services. Chronic Conditions. Chronic Conditions Data Warehouse. Accessed November 14, 2024. https://www2.ccwdata.org/condition-categories-chronic

4. Rural Health Research Center. Rural Urban Commuting Area Codes Data. Accessed November 14, 2024. https://depts.washington.edu/uwruca/ruca-uses.php

© 2025 Zhou FF et al. *JAMA Network Open*.
